# Tonic stimulation of the pharyngeal mucosa causes pain and a reversible increase of inflammatory mediators

**DOI:** 10.1007/s00011-013-0663-7

**Published:** 2013-09-15

**Authors:** Bertold Renner, Gabi Ahne, Elke Grosan, Birgit Kettenmann, Gerd Kobal, Adrian Shephard

**Affiliations:** 1 Department of Experimental and Clinical Pharmacology, University of Erlangen-Nuremberg, Krankenhausstr. 9, 91054 Erlangen, Germany; 2 Altria Client Services Inc., Richmond, VA USA; 3 Reckitt Benckiser Healthcare Ltd, Slough, UK

**Keywords:** Cold dry air, Experimental model, Inflammation, Sore throat, Pharyngitis

## Abstract

**Objective and design:**

To develop a model of the inflammatory component of non-infectious sore throat using tonic stimulation and quantification of inflammatory mediators in pharyngeal lavage fluid.

**Material or subjects:**

Forty-five healthy volunteers.

**Treatment:**

Cold dry air.

**Method:**

Tonic stimulation of the pharynx was achieved using a constant stream of cold dry air to the back of the throat. Following optimization of stimulation conditions (phase 1), pharyngeal pain, irritation, and swallowing discomfort were assessed using visual analog scales, and the concentration of inflammatory markers were measured in pharyngeal lavage fluid (phase 2).

**Results:**

Optimum conditions for tonic pharyngeal stimulation were cold dry air at 12 °C, relative humidity 20 %, at a flow rate of 12 L/min for 15 min. Analysis of pharyngeal lavage fluid collected 5 min after stimulation showed significant increases in prostaglandin E_2_ (*P* = 0.018), thromboxane B_2_ (*P* < 0.001), and substance P (*P* < 0.001), but no increase in peptidoleukotriene. When the stimulus was removed, the level of inflammatory markers in pharyngeal lavage fluid returned to baseline by 30 min post-stimulation. These objective measures mirrored subjective pain ratings.

**Conclusions:**

Tonic stimulation of the pharyngeal mucosa with cold dry air causes pain and an increase of inflammatory mediators which are reversible.

**Electronic supplementary material:**

The online version of this article (doi:10.1007/s00011-013-0663-7) contains supplementary material, which is available to authorized users.

## Introduction

Pharyngitis (sore throat) can be caused by infectious agents (viruses, bacteria, and fungi) or physico-chemical (such as smoking, snoring, shouting, tracheal intubation, medications, or concomitant illness) or environmental (including indoor and outdoor air pollutants, temperature and humidity, and hazardous or occupational irritants) factors [1]. However, the underlying pathophysiology of non-infectious sore throat is not well understood, and experimental models with defined conditions and objective endpoints are needed to study the mechanisms and evaluate treatment strategies [1].

Many of the currently available models are not specific for sore throat or use subjective endpoints. There are several models for the study of non-allergic rhinitis, but they mainly induce nasal symptoms—examples include intranasal challenge with histamine [2], capsaicin [3], or methacholine [4], as reviewed previously [5]. While nasal provocation with bradykinin induces sore throat, it also induces rhinitis [6]. The rating of sore throat pain [7, 8] is specific for sore throat, but the endpoint is subjective. In preclinical settings, there is no animal model available to test drugs for the treatment of sore throat.

Nasal stimulation with cold dry air is an established model for rhinitis [5], and may be a suitable model for the inflammatory component of some etiologies of non-infectious sore throat when applied to the pharynx. When introduced to the nasal cavity of volunteers, a stream of air induces pain, the intensity of which varies depending on humidity and temperature [9], and potentially air flow rate. Previous work in our laboratory used cold dry air (22 °C, 20 % relative humidity, 8 L/min) to experimentally induce rhinitis in healthy volunteers [10]. The assessment of inflammatory mediators in nasal lavage fluid provides an objective measure of inflammation [11] and quantifies the response to nasal cold dry air provocation [10]. Pain induced by nasal application of cold dry air has been used to assess the anti-inflammatory and analgesic properties of acetaminophen, ibuprofen, ketoprofen, and azapropazone [12–15].

Pharyngeal lavage has been used to investigate mucosal inflammation in the pharynx of patients with sleep-related disordered breathing [16]. In the current study, we developed a model of pharyngeal inflammation using local application of cold dry air, with quantification of inflammatory mediators in pharyngeal lavage fluid.

## Materials and methods

The study was approved by the University of Erlangen-Nuremberg’s institutional review board, and was conducted in agreement with the Declaration of Helsinki (Tokyo amendment). Parts of the data are published in a thesis [17].

### Subjects

A total of 45 healthy volunteers participated, with a mean age of 25.2 ± 1.9 years (range 22–34 years), including 23 males and 22 females. Five subjects participated in preliminary experiments. Subsequently, 20 subjects participated in phase 1 and 20 subjects participated in phase 2. They were recruited at the Department of Experimental and Clinical Pharmacology and Toxicology, University of Erlangen-Nuremberg, Germany. The study was conducted between October 1998 and June 2000, and prior to commencement, written informed consent was obtained from all subjects. The participants received 100 DM (about 50 Euro) for each experimental session.

Inclusion criteria included volunteers who were physically and mentally healthy, aged 18–45 years, and of normal body weight (Broca Index ±25 %). Excluded were those subjects with evidence or suspicion of any clinical abnormality, any acute or chronic infection or allergy requiring therapy, those who were taking concomitant medication (except contraceptives) or who had taken medication within the previous 4 weeks, and those with relevant loss of blood within the last month. Smokers (more than 15 cigarettes/day) were excluded, as were people dependant on drugs or ingesting more than 60 g alcohol/day, and those with any diet (including vegetarian) or lifestyle that would interfere with the study. Also excluded were people with known or suspected non-compliance, pregnant or lactating women, and those with actual or anamnestic bronchial asthma.

All subjects underwent pre-study screening to confirm their health, including a general medical examination, measurement of vital signs, and 12-lead electrocardiogram.

In a training session prior to the study, the subjects were familiarized with the experimental procedures and with velopharyngeal closure, a breathing technique that avoids respiratory flow within the nasal cavity during stimulation [18]. This ensures the delivery of the stimulus is independent of breathing through the nose which could otherwise influence its intensity and hence the subject’s response.

### Tonic stimulation of the pharynx

**Fig. 1:**
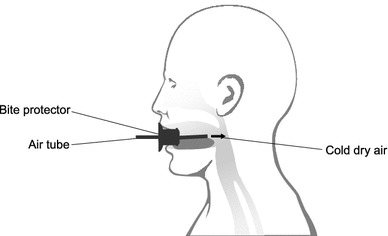
Schematic diagram showing the apparatus used to apply cold dry air to the pharynx of volunteers

Stimulation of the pharynx was achieved using a constant stream of cold dry air to the back of the throat (Fig. 1). The optimum conditions for this were established in phase 1. Low relative humidity (20 %) was achieved by passing the airstream through a bottle filled with silica gel. For thermostabilization, the bottle and tubing were located in a thermostat. All materials were made of glass or Teflon^®^.

During all studies, the subjects were comfortably seated in an air-conditioned room with white noise used to provide acoustic shielding. Subjects fasted for at least 6 h before each experiment. They brushed their teeth 2 h before each experiment with the standard toothbrush and toothpaste provided. Thereafter, no smoking and only water was allowed. Subjects cleaned their mouth with water 15 min before each session.

In order to stabilize vigilance during the experiments, the subjects were required to perform a tracking task on a video screen [19]. Using a joystick, they had to keep a small square within a randomly moving larger square. By measuring this ‘tracking performance’ it was possible to monitor the vigilance of the subjects.

### Phase 1: optimization of tonic pharyngeal stimulation

A range of conditions for stimulation of the pharynx were investigated in pilot experiments in five subjects. Optimum conditions for stimulation of the pharynx were identified in phase 1 of the study in 20 subjects: at a relative humidity of 20 %, different combinations of air flow, temperature, and duration of stimulus were investigated (Table 1). Pain intensity and aversion were assessed by a visual analog scale (VAS) throughout stimulation. Each subject underwent 15 sessions (randomized, single blind) in total (Table 1), with one session per day and at least 2 days between each session.

**Table 1:** The conditions which were investigated during phase 1 to optimize pharyngeal stimulation, at 20 % relative humidity

Condition	Air flow (L/min)	Temperature (°C)	Duration of stimulus (min)
1	12	12	15
2	12	12	5
3	12	12	10
4	12	12	20
5	12	12	25
6	6	12	15
7	8	12	15
8	10	12	15
9	14	12	15
10	12	4	15
11	12	6	15
12	12	8	15
13	12	10	15
14	12	15	15
15	12	18	15

### Phase 2: effect of tonic pharyngeal stimulation on inflammatory markers and pain

In phase 2, the effect of the optimum cold dry air conditions determined in phase 1 (12 °C, relative humidity 20 %, at 12 L/min for 15 min) on pharyngeal pain, irritation, and swallowing discomfort (VAS) and concentration of inflammatory markers in pharyngeal lavage fluid was assessed in 20 subjects. Each subject underwent one session of tonic pharyngeal stimulation on each of 2 days.

### Assessment of subject’s perception of pain and discomfort

*During stimulation*, every 60 s during the application of the air flow to the pharynx, the subjects rated the intensity of and aversion to pain on a VAS displayed on a computer screen [19]. The pain intensity scale ran from 0 (no pain) to 100 (maximal imaginable pain) and the aversion scale ran from 0 (no aversion) to 100 (maximal aversion). The pain intensity scale was the primary indicator, whilst the aversion scale was used to check for non-pain adverse events or discomfort.

*After stimulation*, throat pain, throat irritation, and discomfort while swallowing were assessed every 10 min for 80–90 min post-stimulation. The VAS for these ran from 0 (no pain) to 100 (maximal imaginable pain), from 0 (no irritation) to 100 (maximal irritation), and from 0 (no swallowing discomfort) to 100 (maximal swallowing discomfort).

### Pharyngeal lavage technique

Pharyngeal lavage was performed immediately before (at 0 min), and at 5 and 30 min after pharyngeal stimulation. The subjects were asked to gargle with 10 mL of warmed (37 °C) Ringer’s solution (pH 5–7) for 10 s. After collecting the lavage fluid it was immediately centrifuged (10 min, 1,700 rpm, 4 °C) to separate the cell pellet, then frozen at −80 °C until further analysis.

### Measurement of inflammatory mediators

Pilot experiments in five subjects determined if inflammatory mediators could be measured in pharyngeal lavage fluid. The concentrations of inflammatory mediators [prostaglandin E_2_ (PGE_2_), peptidoleukotriene (PLT), thromboxane B_2_ (TXB), and substance P (SP)] in pharyngeal lavage fluid were measured by enzyme-linked immunosorbent assay (ELISA) (Cayman Chemical Company, Ann Arbor, MI, USA). These mediators were selected following their responsiveness to nasal stimulation with cold dry air [10], and the presence of substance P in sensory nerve fibres of the upper respiratory tract [20]. After defrosting, analysis of TXB and SP was conducted using undiluted samples, whilst those for PGE_2_ and PLT were diluted 1:10 with Ringer Lactate solution (Braun, Melsungen, Germany). The intensity of colour change was determined spectrophotometrically (microplate reader MR2100, Firma Dynex Technologies, Düsseldorf, Germany). Results outside the validated range were excluded and treated as missing data.

### Statistical analyses

Outcome measures were subjective ratings of pain intensity and aversion during stimulation, subjective ratings of pain, irritation, and swallowing discomfort after stimulation, and pre- and post-stimulation concentrations of inflammatory mediators (PGE_2_, PLT, TXB, and SP) in lavage fluid.

All statistical analyses were conducted using IBM SPSS Statistics 21 (Chicago, IL, USA). Descriptive analyses included calculation of mean, range, standard deviation, and standard error. Repeated measures ANOVA was used for inflammatory mediators in order to detect stimulation and recovery effects over time. Student’s *t* tests were used as post hoc tests, adjusting each *P* value according to Bonferroni. Spearman rank correlation coefficient was used for analysis of correlations between increases of different mediators and pain ratings after cold dry air stimulation compared with baseline. Data are presented as means with 95 % confidence intervals (CI). *P* < 0.05 was considered significant.

## Results

### Phase 1: optimization of tonic pharyngeal stimulation

**Fig. 2:**
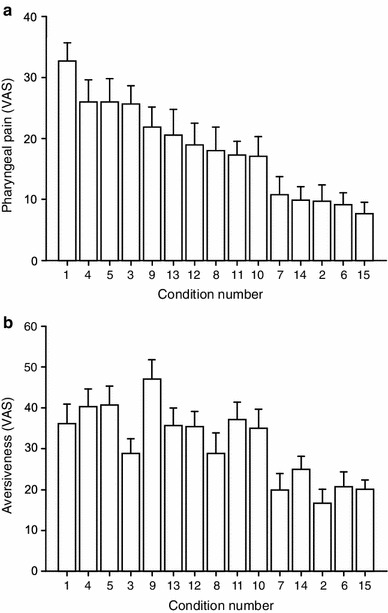
Pharyngeal pain intensity (**a**) and aversion (**b**) (mean 95 % confidence interval), measured on visual analog scales (VAS), during phase 1 tonic pharyngeal stimulation with cold dry air varying in flow rate, temperature, and duration of stimulus. For details of the air flow characteristics for each of the 15 experiments see Table 1

When combinations of stimulation parameters (Table 1) were investigated in phase 1 (*n* = 20) it was clear that the greatest intensity of pharyngeal pain was achieved using 12 L/min, at 12 °C, for 15 min (condition 1 in Table 1; Fig. 2a). Warming the air to 18 °C considerably reduced the pain intensity (condition 15 in Table 1; Fig. 2a). Cooling the airflow to below 12 °C (conditions 10–13) or increasing the duration of the stimulus (conditions 4–5) did not further increase the pain intensity (Fig. 2a). Ratings of aversion throughout generally followed the same trends (Fig. 2b). The optimum conditions for tonic pharyngeal stimulation were therefore determined to be 12 L/min at 12 °C for 15 min.

### Phase 2: effect of tonic pharyngeal stimulation on inflammatory markers and pain

**Fig. 3:**
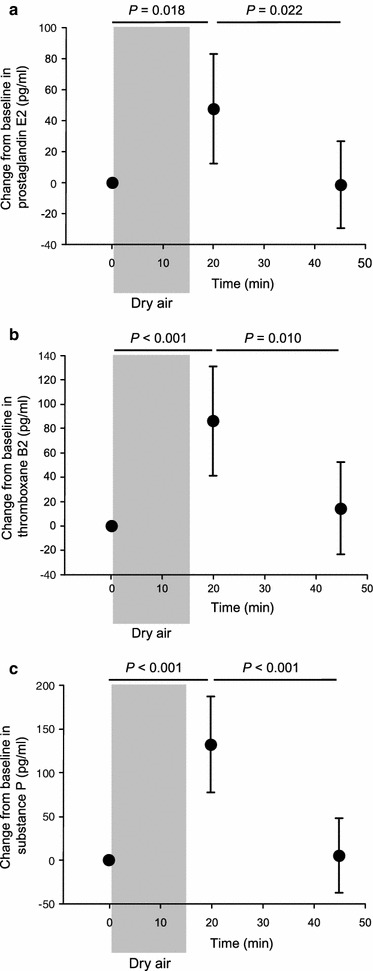
Change (mean 95 % confidence interval, *n* = 20) in **a** prostaglandin E2, **b** thromboxane B2, **c** substance P in pharyngeal lavage fluid of healthy volunteers after tonic pharyngeal stimulation (the grey shaded area) with cold dry air (12 °C, 20 % humidity at 12 L/min for 15 min). Results are the means of two tests. Student’s *t* test was used with Bonferroni-adjusted *P* values shown

In phase 2 (*n* = 20), there was a significant change in all inflammatory mediators over time (PGE_2_: *df* = 2, *F* = 5.7, *P* = 0.005; TXB: *df* = 2, *F* = 8.5, *P* < 0.001; SP: *df* = 2, *F* = 18.5, *P* < 0.001, PLT: *df* = 2, *F* = 4.4, *P* = 0.028). Post hoc analysis of pharyngeal lavage fluid collected 5 min after tonic pharyngeal stimulation showed significant increases in PGE_2_ (*P* = 0.018), TXB (*P* < 0.001), and SP (*P* < 0.001) compared with baseline (Fig. 3). When the stimulus was removed, the level of inflammatory markers in pharyngeal lavage fluid returned to baseline by 30 min post-stimulation (Fig. 3). There was no significant increase in PLT upon tonic pharyngeal stimulation (change from baseline 161 pg/mL (95 % CI 52–270 pg/mL) and 105 pg/mL (95 % CI 10–200 pg/mL) for first and second time point after stimulation, *n* = 14).

**Fig. 4:**
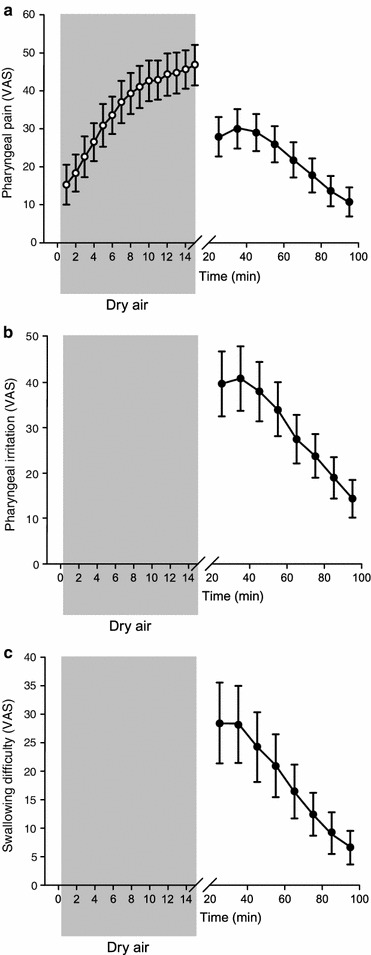
Change (mean 95 % confidence interval, *n* = 20) in pharyngeal pain intensity (**a**), irritation (**b**) and swallowing difficulty (**c**) of healthy volunteers, measured on visual analog scale (VAS), during (the grey shaded area) and after tonic pharyngeal stimulation with cold dry air (12 °C, 20 % humidity at 12 L/min for 15 min). Results are the means of two tests. VAS for pharyngeal irritation and swallowing difficulty were not recorded during the stimulation period

The phase 2 VAS results (*n* = 20) show the pharyngeal pain (Fig. 4a) and stimulus aversion (Fig. 4d, supplement) during tonic pharyngeal stimulation (12 L/min at 12 °C for 15 min), which increases progressively from the onset of the stimulus and throughout its duration. After removal of the stimulus, irritation (Fig. 4b) and swallowing difficulty (Fig. 4c) peak and then progressively improve over the next 80–90 min, and pain reduces to baseline (Fig. 4a).

Correlation analysis showed that the increased pain ratings during cold dry air stimulation and decreased ratings thereafter coincided with changes in mediator release. This was significant for TXB (*P* = 0.042; *r* = 0.231) and SP (*P* = 0.034; *r* = 0.237), but was not significant for PLT and PGE_2_.

## Discussion

This study shows that tonic stimulation of the pharyngeal mucosa with cold dry air causes pain, irritation, and discomfort whilst swallowing and an increase of inflammatory mediators, which is reversible. This is the first sore throat model that is both specific to the pharynx and includes an objective endpoint (pharyngeal inflammatory markers). Other currently available techniques [7, 8] do not routinely measure inflammatory mediators and rely on assessment of the physical appearance of inflammation.

The properties of an ideal pain model have previously been described [21]. An ideal stimulus should exhibit minimal neurohistologic variation between individuals, should be measureable, closely associated with the changes which produce inflammation, provoke minimal tissue damage, show a relation to pain intensity, provide information about discrimination between stimuli, result in repeatable stimulation without temporal interaction, be applied easily, allow a quantifiable determination of the quality of inflammation, be sensitive to agents of low analgesic power, show dose-related effects of anti-inflammatory drugs, and be applicable for both man and animal [10]. The current sore throat pain model satisfies many of these criteria, although its sensitivity and dose-response to anti-inflammatory agents was not tested.

The optimum conditions for pharyngeal stimulation were confirmed to be cold dry air at 12 L/min, 20 % relative humidity and 12 °C, with a 15-min stimulus duration. These conditions maximized the subjective pharyngeal pain reported by the volunteers, and hence maximized the sensitivity of the model. The conditions required for optimum tonic stimulation of the pharynx are somewhat harsher than those employed for the nasal cavity (22 °C, 20 % relative humidity, 8 L/min) [10], and this may be due to the relative sensitivity of the mucosa at these locations. There may be differences in innervation and/or neuropeptide release [20], and the clearance of inflammatory mediators and neuropeptides on the pharyngeal mucosa may also be enhanced by saliva production, which is not the case in the nasal cavity. The single-layer respiratory epithelium (pseudostratified) changes to a multiple-layer epithelium in the lower pharynx, and this could also influence sensitivity.

The increase in inflammatory markers in pharyngeal lavage fluid upon tonic stimulation with cold dry air provides an objective measure that mirrors the subjective pain ratings. Both pain and inflammatory markers increased upon stimulation then returned to baseline shortly afterwards. The timing of the peak of inflammatory markers coincided with the pain measures. However, inflammatory mediators returned to baseline more quickly (at about 30 min after the stimulus was removed) compared with pain measures (at about 80–90 min after the stimulus was removed). In this regard, we could not exclude any dilution effects caused by repetitive lavage sampling, which will be pronounced during post-stimulation periods. Our analysis reflected this time shift as only SP and TXB but not PGE_2_ or PLT release correlated with changes in pain ratings. However, a moderate correlation is typical for subjective ratings and was also observed in a previous study, following stimulation of the nasal mucosa with cold dry air [22].

In the current study, PGE_2_, TXB, and SP levels in pharyngeal lavage fluid were significantly increased by cold dry air; but there was no significant effect on PLT. In contrast, a previous study found significant increases in PLT in nasal lavage fluid when cold dry air was applied to the nasal cavity, although increases in PGE_2_ and TXB failed to reach significance [10]. The differences between studies may be due to variability in the data or missing values. The data are currently insufficient to determine if the differences in mediator responses (within the current study, and between studies) are due to anatomical location, stimulus characteristics, or other methodological variation. Previous data on pharyngeal lavage are not informative as they are limited to inflammatory cell counts rather than inflammatory mediators [16]. The significant increases in pharyngeal PGE_2_, TXB, and SP in the current study show that these mediators are implicated in the inflammation induced by cold dry air. Whereas SP is known to be involved in nociception and the development of hyperalgesia, PGE_2_ and TXB may have contrary physiological effects (for example, bronchial relaxation versus constriction). We included these mediators because PGE_2_ and TXB may be used to quantify the effect of selective and unselective cyclooxygenase inhibitors [23]. As we measured the acute release of these mediators (during the 15-min stimulation period), there is limited information about lipid mediator production which takes 30 min or longer. However, since we observed rapid recovery of pain and mediator release, the stimulation could potentially be repeated several times per day in order to obtain longer-term effects on inflammatory mediator induction or regulation. Other lipid mediators are likely to be involved but were not analyzed in the current study. Regulators of pharyngeal inflammation (pro-inflammatory and anti-inflammatory) warrant further study, and the current model may provide a tool for this. From human studies of nasal challenge with cold dry air there is a clear suggestion of generation of leukotrienes and kinins; although only in predisposed subjects with rhinitis [22, 24].

The lack of PLT response in the current study may be due to a higher degradation of PLT or greater differences in local production/secretion of PLT. The ELISA kit used for analysis of PLT in the current study detected leukotriene (LT) C_4_, LTD_4_ and the degraded metabolite LTE_4_. Since the kit provides a high specificity for LTC_4_ and LTD_4_ (100 %) and a lower specificity for LTE_4_ (below 70 %), differences in degradation state may contribute to the PLT data variability observed in the current study. The degradation of LTE_4_ to undetectable metabolites is incomplete in bronchoalveolar samples, but the degradation time response is currently unknown in samples containing saliva. Methodological differences between the current study and the nasal lavage study [10] include different sample volumes (10 vs. 6 mL, respectively), which could affect sensitivity.

In summary, the cold dry air model presented here provides a well-controlled, easily-applied technique for inducing reversible sore throat pain that is specific to the pharynx and can be measured objectively. This model will be of benefit for the future development of analgesics for alleviating non-infectious sore throat of some etiologies with an inflammatory component, the investigation of environmental causes of sore throat [1] including allergic and nonallergic states in environmental medicine, as well as toxicology exposure studies with defined stimulus conditions in addition to cold dry air.

## Electronic supplementary material

Below is the link to the electronic supplementary material.

Supplementary Fig. 4d for reviewers or perhaps supplement, showing aversion during stimulation. Change (mean 95 % confidence interval, *n* = 20) in stimulus aversion ofhealthy volunteers, measured on visual analog scale (VAS), during (the *greyshaded area*) tonic pharyngeal stimulation with cold dry air (12 °C, 20 % humidityat 12 L/min for 15 min). Results are the means of two tests. VAS for stimulusaversion were not recorded during the recovery period (EPS 610 kb)
